# Live Tissue Imaging to Elucidate Mechanical Modulation of Stem Cell Niche Quiescence

**DOI:** 10.5966/sctm.2015-0306

**Published:** 2016-07-28

**Authors:** Nicole Y.C. Yu, Connor A. O'Brien, Iveta Slapetova, Renee M. Whan, Melissa L. Knothe Tate

**Affiliations:** ^1^ Graduate School of Biomedical Engineering University of New South Wales, Sydney, Australia; ^2^ Biomedical Imaging Facility, Mark Wainwright Analytical Centre, University of New South Wales, Sydney, New South Wales, Australia

**Keywords:** Periosteum‐derived stem cells, Stem cell niche quiescence, Tissue mechanical prestress, Confocal microscopy, In situ tissue imaging, Stem cell mechanics

## Abstract

The periosteum, a composite cellular connective tissue, bounds all nonarticular bone surfaces. Like Velcro, collagenous Sharpey's fibers anchor the periosteum in a prestressed state to the underlying bone. The periosteum provides a niche for mesenchymal stem cells. Periosteal lifting, as well as injury, causes cells residing in the periosteum (PDCs) to change from an immobile, quiescent state to a mobile, active state. The physical cues that activate PDCs to home to and heal injured areas remain a conundrum. An understanding of these cues is key to unlocking periosteum's remarkable regenerative power. We hypothesized that changes in periosteum's baseline stress state modulate the quiescence of its stem cell niche. We report, for the first time, a three‐dimensional, high‐resolution live tissue imaging protocol to observe and characterize ovine PDCs and their niche before and after release of the tissue's endogenous prestress. Loss of prestress results in abrupt shrinkage of the periosteal tissue. At the microscopic scale, loss of prestress results in significantly increased crimping of collagen of periosteum's fibrous layer and a threefold increase in the number of rounded nuclei in the cambium layer. Given the body of published data describing the relationships between stem cell and nucleus shape, structure and function, these observations are consistent with a role for mechanics in the modulation of periosteal niche quiescence. The quantitative characterization of periosteum as a stem cell niche represents a critical step for clinical translation of the periosteum and periosteum substitute‐based implants for tissue defect healing. Stem Cells Translational Medicine *2017;6:285–292*

**Significance Statement**

Previous studies have shown a significant correlation of periosteum‐derived stem cell tissue genesis with mechanical cues imbued through subtle physiological loading such as stance shift after surgery. How a change in baseline mechanical stress state can possibly be transduced to mesenchymal stem cells residing in the periosteal niche is a major conundrum in the field. A novel platform to integrate cutting‐edge live cell and tissue imaging technology with the current understanding of stem cell‐mediated tissue genesis and healing is described. The loss of baseline prestress intrinsic to periosteal tissue in a healthy, normal state results in an immediate and persistent shrinkage of tissue at a macroscopic scale that correlates with changes in collagen crimping and the number of rounded cell nuclei in the cambium layer of the tissue. Given a body of work demonstrating the link between stem cell and nucleus shape and lineage commitment, these results are consistent with a direct transduction of mechanical signals from a tissue to cellular length scale. These insights could have profound implications for mechanical regulation of periosteal stem cell niche quiescence.

## Introduction

The periosteum, a composite cellular and connective tissue, bounds all nonarticular bone surfaces and provides a niche for mesenchymal stem cells (MSCs) 1 2 3. Hyperelastic periosteal tissue exhibits a remarkable regenerative capacity; in critical size long bone defects surrounded in situ by periosteum, periosteum‐derived cells (PDCs) egress from the tissue sheath within days of injury to regenerate tissue, infilling the defect with intramembranous woven bone within weeks 4 or endochondrally produced bone within months 5 6 7 8. This process is modulated by both PDCs and periosteum's endogenous extracellular matrix (ECM) proteins 5, 6. Mechanical loading has been shown to correlate with an increase in periosteum's tissue regeneration capacity 9, 10. Mechanobiology has been hypothesized to be a key modulator of periosteal stem cell niche quiescence; hence, an understanding of the mechanobiology of the periosteal niche is expected to facilitate use of the tissue and its resident cells in a translational context 7, 10 11 12 13.

Cells isolated from human periosteal tissue, referred to as periosteum‐derived cells (PDCs), exhibit MSC surface markers, including CD73, CD90, and CD105 3, 12. Additionally, human PDCs express both epithelial and mesenchymal junctional adhesion proteins, providing a template for tissue architecture and playing a role in molecular mechanisms of chondro‐, osteo‐, and adipogenesis 8. Although more PDCs reside in the inner cambium layer of the periosteum compared with the fibrous layer 1, 2, 7, 8, 14 15 16 17, greater than 99% of PDCs isolated through enzymatic digestion (encompassing cells from the fibrous and cambium layers both) and/or migration protocols (preferentially cells migrating from the cambium layer) exhibit MSC surface markers, including CD73, CD90, and CD105. In contrast, slightly less but more than 95%, 97%, and 95% of bone marrow stromal cells from human patients exhibit the MSC surface markers CD73, CD90, and CD105, respectively 3.

Periosteum and periosteum‐inspired surgical membrane implants have been used to heal critical size bone 3, 5, 9, 10, 13, 18 and cartilage defects 19 20 21 22 23 in preclinical animal models and clinical (human) cases 24 25 26 27 28 29. Numerous small animal studies have demonstrated the importance of the periosteum for bone healing 29, 30. Removal of the periosteum has been shown to significantly reduce bone healing 30 31 32 33, and murine cell tracing experiments demonstrated that up to 90% of intramembranous and endochondral bone defect healing is derived from the periosteum 29. A series of ovine studies demonstrated that retention of the periosteum in situ around a hematoma‐filled defect is not only sufficient but also efficient for healing critical size femoral defects 5, 9, 10, 13, 18. In vitro, progenitor cells from human periosteum exhibit the capacity for chondro‐, osteo‐, or adipogenesis, similar to or exceeding that of human bone marrow stromal cells. In these studies, cells were isolated either through trypsinization or by collection after egression from the cambium side of the sample 2, 3.

A key to translating periosteum's regenerative capacity is an understanding of the mechanical cues that trigger PDCs to egress and home to sites of injury. The outer fibrous layer of the periosteum is composed of the extracellular matrix structural proteins, collagen and elastin, which, respectively, impart toughness and elasticity to the tissue. Collagenous Sharpey's fibers anchor the periosteum to bone surfaces 1, 2, 7, 14 15 16 17. Periosteum exhibits prestress in situ, comprising approximately 12.1 ± 0.40 MPa in the longitudinal direction and 0.77 ± 0.43 MPa in the circumferential direction of the adult ovine femur 7. In parallel, periosteum exhibits anisotropic mechanical properties, with significantly higher elastic modulus in the longitudinal than in the circumferential direction. On separation of Sharpey's fibers due to periosteal lifting or resection, the stress state of the tissue changes abruptly, and the tissue shrinks anisotropically 33. The subsequent rapid mobilization of periosteal MSCs to areas of injury and PDCs' genesis of repair tissue correlate significantly with the abrupt change in the periosteum's stress state 9, 4, 18. Mechanical loading has also been shown to correlate with the altered patterns of gene expression associated with increased PDC proliferation 34.

A body of work in stem cell mechanics has pointed increasingly to a role for biophysical cues in modulating stem cell shape and fate 35 36 37 38 39. In the context of these studies of periosteal mechanobiology and regeneration, our working hypothesis was that changes in the periosteum's baseline stress state would modulate the quiescence of its stem cell niche by altering the fate of the inhabitant PDCs. Our approach was to develop a live three‐dimensional (3D) cell and tissue imaging protocol to study periosteal tissue and its inhabitant cell population and the changes in the same associated with the loss of tissue prestress. We hypothesized that the loss of prestress would result in changes to both periosteum's fibrous tissue architecture and its inhabitant PDC population.

## Materials and Methods

Live cell and tissue imaging studies were performed to observe microscopic changes related to the loss of prestress in an ovine model. The rationale for the choice of the ovine model was twofold. First, we aimed to build on our previous body of work using the ovine model and its translation from in vitro 3, 33, 40 to ex vivo 10 to in vivo 4 5 6, 11, 13, 18 to clinical studies 3, 24, 25. Also, in contrast to the few cell layer‐thick murine periosteum 29, the ovine femoral periosteal thickness approximates that of humans 7, 17. Specifically, measures of the ovine femur periosteum (i.e., 160.0 ± 23 µm 7, 17, 39, 40) are similar to those of the human femoral periosteum thickness (i.e., 100 μm in patient donors aged 68–99 years) 2, 3, 17. The periosteum thickness decreases significantly with age and sample site. For example, the mean thickness of the ovine tibial periosteum is approximately 417 µm at 2 years of age and 371 µm at 3 years of age. The mean thickness of the femoral periosteum in the older cohort is significantly smaller than that of the tibia from the same cohort 7.

Two‐photon confocal microscopy was used for live and direct three‐dimensional visualization of the cells and collagen structure within the periosteum. A cell‐permeant fluorescent probe was selected to mark nucleic acids within the nucleus (DNA) and thereby identify cell nuclei instead of entire cells, facilitating identification of individual cells and easing cell count quantification. Two‐photon confocal microscopy with second harmonics generation was used for collagen imaging without the need for freezing, cutting, or other techniques that could change the collagen structure of the tissue.

### Periosteum Sample Harvest

**Figure 1:**
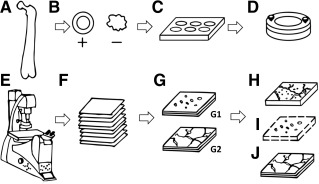
Methodology flowchart for elucidating the periosteal stem cell niche using confocal live cell imaging. **(A):** Isolation of ovine femur. **(B):** Resection of periosteum samples with prestress (+) or no prestress (−). **(C):** Sample tissue culture and fluorescent staining. **(D):** Placement of sample in chamber. **(E):** Live cell and tissue confocal imaging. **(F):** Collection of image stacks. **(G):** Image processing of separate image channels (cell nuclei **[G1]** and collagen **[G2]**). **(H):** Three‐dimensional (3D) reconstruction of merged image stacks. **(I):** 3D cell counting. **(J):** Reconstruction of collagen with 3D image stacks.

Periosteum samples were harvested from the femora of male wether (castrated) sheep (*n* = 3). The sheep were harvested under the authority of the University of New South Wales Australia Animal Ethics Committee. Periosteal samples were resected and prepared for study following a modified, previously published protocol 40. In brief, the skin and overlying muscle were removed to expose the femur under sterile conditions (Fig. 1A). The periosteum was carefully cleaned of muscle and tendon insertions using tweezers and a no. 10 surgical blade (Cincinnati Surgical Co., Cincinnati, OH, http://www.cincinnatisurgical.com). The femur and bounding periosteum were regularly saturated with Hartmann's solution (Baxter, Deerfield, IL, http://www.baxter.com) to retain moisture. Tissues (Fig. 1B) were isolated from the femur's medial and lateral aspect of the anterior diaphysis. To maintain in situ prestress (prestress group), samples were affixed to an annular, flexible plastic template (inner diameter, 10 mm; outer diameter, 20 mm; model no. PP2500; 3M, Maplewood, MN, http://www.3m.com) using ethyl 2‐cyanoacrylate glue (Loctite 435, medical grade; Henkel Australia, Kilsyth, VC, Australia, http://www.henkel.com.au) in accordance with previous protocols 40. This allowed for tissue removal while maintaining in situ tissue length, width, and prestress state. A surgical blade was then used to cut along the outer edge of the plastic template, and periosteal elevators (model nos. 399.360 and 399.370; DePuy Synthes, West Chester, PA, http://www.synthes.com) were used to gently separate the Sharpey fibers from the underlying bone and resection of the periosteum. In the periosteum with no prestress (no prestress group), the circular plastic template was used as a guide, and sample resection followed the protocol for the prestress group, with the exception that the samples were allowed to shrink freely. A total of 8 samples were harvested per femur (i.e., *n* = 4 per group per sheep; Table 1).

**Table 1:** Experimental groups

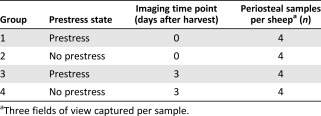

### Periosteum Sample Tissue Culture and Fluorescent Staining

Freshly isolated periosteum samples were directly transferred to tissue culture conditions in a humidified incubator at 37°C with 5% CO_2_ in accordance with previously published protocols 3, 5. The samples were stained fluorescently at set time points, either immediately after harvest or 3 days after harvest (Fig. 1C). In brief, the resected samples were transferred to 12‐well tissue culture plates (CELLSTAR; Greiner Bio‐One GmbH, Frickenhausen, Germany, http://www.greinerbioone.com) with the periosteum sample's cambium facing down. Each tissue culture well contained 2 ml of α‐minimum essential medium (α‐MEM) with GlutaMAX supplemented with 10% fetal bovine serum (FBS) (Thermo Fisher Scientific Life Sciences, Waltham, MA, http://www.thermofisher.com), 1% antibiotic‐antimycotic (Thermo Fisher) overnight. Media were then changed to 2 ml of standard culture medium (α‐MEM with GlutaMAX supplemented with 10% FBS, 1% penicillin‐streptomycin; Thermo Fisher). The standard culture medium was replaced every 2–3 days.

The samples were imaged at two set time points (D_0_, immediately after harvest, and D_3_, 3 days after harvest). Specifically, group 1 and 2 samples were imaged at D_0_ and group 3 and 4 samples were imaged at D_3_. At the set time point (D_0_ or D_3_), the tissue samples were stained with fluorescent Hoechst 33342, trihydrochloride, trihydrate, 0.5 µg/ml prepared in standard culture medium (Thermo Fisher), a cell‐permeant nuclear counterstain that emits fluorescence when bound to double‐stranded DNA 41. The samples were incubated in 2.5 ml of fluorescent stain mixture solution for 1.5 hours in a humidified incubator (37°C with 5% CO_2_), washed with standard culture medium (3 washes, 10 minutes per wash), and stored in standard culture medium until immediately before confocal imaging, at which point the samples were washed in PBS (3 washes, 10 minutes per wash).

### Live Tissue Confocal Imaging

Each freshly stained periosteal sample was placed in a dual flow chamber (Warner Instruments, Hamden, CT, http://www.warneronline.com) with the cambium layer facing down and held between two glass cover slips (15 mm in diameter) and two silicon gaskets (Warner Instruments; Fig. 1D). For the prestressed groups (groups 1 and 3), each periosteum sample and annular template was placed in a dual flow chamber. Confocal imaging (TCS SP5 II multiphoton confocal laser scanning microscope with an HC PL Fluotar 20 × 0.50 NA dry objective; Leica Microsystems, Buffalo Grove, IL, http://www.leicamicrosystems.com) and multiphoton imaging (Mai Tai DeepSee Ti:Sapphire laser, 840 nm; Spectra Physics, Mountain View, CA, http://www.spectra-physics.com) enabled imaging of cell nuclei. The signal from the fluorescent dye Hoechst 33342 was collected in reflected non‐descanned detectors (NDDs) fitted with emission filters of 460/50 nm. In addition, collection of the forward propagating second harmonics generation signal capture was achieved using transmitted NDDs with a 420/20‐nm bypass filter. This enabled imaging of collagen in the periosteum (Fig. 1E). Three‐dimensional stacks were acquired from the periosteal cambium to fibrous layers at 1.97‐μm intervals (Fig. 1F), with signals representing cell nuclei and collagen (Fig. 1G). A total of three fields of view (310 μm × 310 μm) were collected per sample.

### Image Processing

Captured image stacks were viewed and processed with ImageJ2, version 1.49 (NIH, Bethesda, MD, http://www.imagej.nih.gov). Three‐dimensional reconstructions were generated using the 3D Viewer plugin (ImageJ2; NIH) to view the cell nuclei in relation to the collagen fibrils within the periosteal tissue (Fig. 1H). To determine the change in the number of cells exhibiting nuclear rounding (with and without prestress), image stacks of the cambium layer containing rounded nuclei‐stained cells were delineated manually by a blinded observer and processed to remove background noise (ImageJ2; NIH), resulting in a binarized stack of clearly delimited particles representing nuclei. The particles were quantified using the BoneJ: Particle Analyzer plugin (NIH) 42 on binarized image stacks. The nuclei number was normalized to the tissue volume (i.e., number of rounded cellular nuclei in three dimensions divided by the cambium tissue volume [nuclei per μm^3^]; Fig. 1I).

The structure of the collagen matrix with and without prestress was observed using the 3D Viewer plugin and as two‐dimensional (2D) images through stacks (ImageJ2; NIH; Fig. 1J). The change in 2D extracellular matrix structure immediately after harvest (D_0_) was quantified as the degree of crimp in collagen filaments *C*, which was previously defined by *C* = (*l_c_* − *l_s_*)/*l_s_* × 100% 43. The curved length of the collagen fiber bundle (*l_c_*) and the length of the straight line (*l_s_*) connecting the ends of the measured fiber bundle 43 followed previously published definitions 43 and were measured in a semiautomated fashion using the NeuroJ plugin (ImageJ2; NIH; supplemental online Fig. 1). Crimp was measured in three volumes of interest (VOIs) for each periosteum sample. For each VOI, a minimum of 10 collagen fibers was analyzed.

### Statistical Analysis

Statistical analysis was performed using Prism (GraphPad, La Jolla, CA, http://www.graphpad.com). Significance was defined as *p* < .05. Cell nucleus rounding data were analyzed using nonparametric statistical tests (Kruskal‐Wallis) with post hoc Mann‐Whitney *U* tests to compare the prestress and no prestress groups and the D_0_ and D_3_ time points.

## Results

**Figure 2:**
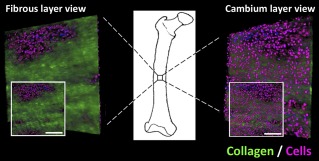
Representative three‐dimensional live tissue image of the periosteal stem cell niche. Depicted three dimensionally from outer fibrous layer (left) and inner cambium layer (right), with two‐dimensional views shown (insets). Collagen indicated by green and nuclei by blue and purple. Scale bars = 100 μm.

**Figure 3:**
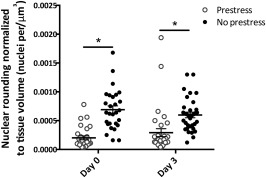
Number of rounded cell nuclei in the cambium of periosteum with and without prestress immediately after harvest (day 0) and 3 days after harvest (day 3), normalized to tissue volume (nuclei per μm^3^). Line represents the median value of the group. ∗, *p* < .01 for comparison with prestress group. Error bars represent SEM.

**Figure 4:**
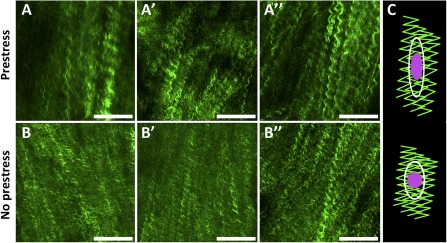
Representative images of collagen in periosteal fibrous layer immediately after harvest with prestress **(A, A′, A″)** and without prestress **(B, B′, B″)**, and schematic of working hypothesis **(C)**. The working hypothesis was that loss of intrinsic tissue prestress and concomitant relaxation in collagen crimping at fibrous layer would lead to cell nucleus shape change (rounding) at the cambium layer. Collagen is represented in green, cell nucleus in purple, and cell boundary in white. Scale bars = 100 μm.

**Figure 5:**
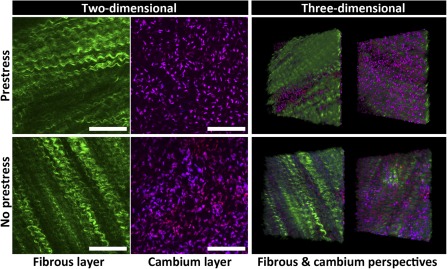
Projections representing the periosteal stem cell niche with prestress (top) and no prestress (bottom) in two and three dimensions. Cell nuclei (purple) and collagen (green) are shown from outer fibrous layer to inner cambium layer of the periosteum. Scale bars = 100 μm.

**Figure 6:**
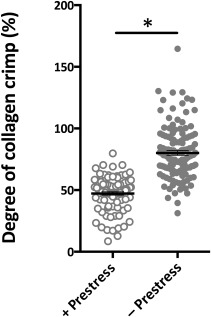
Degree of collagen crimp between samples with and without prestress immediately after harvest. In samples maintaining prestress, significantly less collagen crimp was observed than in samples without prestress. Line represents the median value of the group. ∗, *p* < .01 for comparison with prestress group. Error bars represent SEM.

Spatiotemporal in situ imaging of cells in their periosteal niche allowed for tracking of cell population changes concomitant with the measurement of changes in the periosteum's biomechanical milieu. Specifically, the live, three‐dimensional tissue imaging method facilitated spatiotemporal in situ imaging of cell populations in their periosteal niche (Fig. 2). Periosteum tissue with prestress or with no prestress were imaged immediately or 3 days after harvest. Rounded cell nuclei in the cambium layer were counted in three dimensions to track the changes in the numbers of cells per volume exhibiting rounded nuclei concomitant with the measurement of changes in the periosteal biomechanical milieu. Periosteum with no prestress showed threefold and twofold higher densities of rounded cells compared with the prestress control group at D_0_ and D_3_ (*p* < .01), respectively (Fig. 3). Furthermore, qualitative observations of changes in crimp length suggested structural changes in the collagen in association with changes to the periosteal prestress state (Fig. 4A, 4B) and with respect to the tissue's cambium and fibrous layers (Fig. 5) immediately after harvest (D_0_). Furthermore, quantitative measurements and qualitative observations of the changes in crimp suggested structural changes in the collagen due to periosteal prestress state change in the tissue's fibrous layers. The release of in situ periosteal prestress (i.e., no prestress) increased the degree of collagen crimp by twofold compared with samples with prestress (*p* < .01; Fig. 6).

## Discussion

Loss in prestress of the periosteum, a consequence of the disruption of periosteum's Sharpey's fibers, resulted in immediate relaxation of the tissue, which was observed macroscopically as shrinkage in the unstressed sample. Under the microscope, tissue relaxation coincided with a significant increase in the degree of collagen crimping within the tissue. These changes in the macro‐ and microscopic architecture of collagen within the fibrous layer corresponded with changes in the number of cells exhibiting rounded nuclei. The development of a protocol to image live cells in situ within the periosteal niche provides a basis from which to unravel the regulation of stem cell niche quiescence and the triggering of stem cell activation.

In the cambium layer, the number of cells exhibiting rounded nuclei increased immediately on removal of prestress from the periosteal tissue, before proliferation or migration of the cells could possibly occur. Hence, these changes are indicative of an immediate change in nuclear shape coinciding with relaxation of prestress in the tissue. The density of cells with rounded nuclei (number of cells per volume) increased by approximately threefold on relaxation of the tissue and persisted after 3 days in culture. Similarly, the number of rounded nuclei in samples with and without prestress did not change significantly from day 1 to day 3 in culture.

The nucleus is a cellular mechanosensor with lamins (A/C and B1/2) that contribute to nuclear‐cytoskeletal coupling and mechanotransduction 44. Recent studies have shown that mechanotransduction mechanisms in the nucleus facilitate dynamic regulation of the nucleoskeleton in response to mechanical stress 45. An increasing body of work on nuclear mechanotransduction has linked changes in nuclear shape and structure with physical forces 37, 38, 45 46 47. Further studies have shown mechanical coupling between the extracellular matrix and the cell nucleus 37, 38, 46 47 48 49. Mechanical stress stimuli can be transmitted through a direct molecular link between the extracellular molecules and the nucleoplasmic compartment 48. Differences in stress transmission between the extracellular matrix and the nucleus also show cell type‐dependent responses in nuclear structures 45.

As highlighted by cell migration studies, temporal nuclear shape changes can be viewed as a “negative image” of the physical space and discontinuities of the extracellular matrix 44. Changes in nuclear shape and structure can lead to conformational changes in chromatin structure and organization that directly changes transcription regulation 37, 38, 46, 47. Furthermore, nucleus shape and structure changes are also strongly related to modulation in cellular function and phenotype in both physiological and pathological situations, especially in a mechanically stimulated tissue environment 47. For example, compression‐induced shape changes in chondrocyte nuclei are associated with changes in cartilage composition and density 50, 51. The shape and size of the nucleus is dependent on the cell type and can vary within cell lines; however, most cells imaged in situ and in 3D culture show ovoid or spherical nuclei with a diameter of 5–15 μm 44, 52. In contrast, 2D culture leads to more spread out cells with disk‐shaped nuclei with a diameter of 10–20 μm and a few microns in height 44, 52. Because nuclear shapes and sizes can vary among extracellular matrix structures 44, 52, 53, our in situ 3D live tissue imaging protocol demonstrates greater physiological relevance compared with 2D culture models.

In the fibrous layer, the degree of collagen crimp increased with release of in situ prestress. Immediately after harvest, the degree of crimp increased by twofold. Previous studies have demonstrated significant tissue shrinkage after resection of periosteum without maintenance of prestress 7, 33. The lack of tension on elastin fibers is expected to contribute to macroscopic tissue shrinking. A similar relationship between tension and collagen crimping is well‐described for tendons 54 55 56 57, with tendon stretching decreasing the collagen crimp number and increasing the crimp angle (i.e., flattening) 54. Our observation of a decrease in crimp length of collagen within the fibrous layer of the periosteum is consistent with these findings.

Our working hypothesis was that nuclear shape change (rounding) in the cambium layer would be related to loss of intrinsic tissue prestress and concomitant relaxation in collagen crimping in the fibrous layer (Fig. 4C). In addition, the observation of changes in collagen architecture in the present study suggests a role for collagen structure in the smart permeability properties of the periosteum. Thus, the permeability increases significantly when prestress is lost 40. Finally, the observed abrupt change in nuclear shape might relate to changes in cell shape, structure, and function 35 36 37 38 39. Such shape changes might provide a mechanism for the epithelial to mesenchymal transition observed with periosteal lifting and resection, in which PDCs stabilized by cell‐cell junction proteins 8 become motile and egress into the hematoma‐filled defect, rapidly proliferating and upregulating gene transcription for secretion of ECM proteins associated with tissue genesis 8, 35. Taken as a whole and given the body of data tying stem cell and nucleus shape and fate 35 36 37 38 39, the rounding of nuclei in association with loss in periosteal prestress might trigger activation of PDCs residing in the cambium layer and loss of quiescence. Ongoing studies have been designed to elucidate the time course of these events.

Although our ultimate goal is to gain and apply these insights to harness the periosteum's regenerative capacity, fundamental studies are needed to understand cell behavior in a controlled yet physiological context. An inherent limitation of the present study was the use of resected ovine periosteum to understand the regulation of stem cell niche quiescence. The current model was designed to mimic conditions from our series of previous ovine studies demonstrating the regenerative potential of patent (intact vascular supply) and resected periosteum, in addition to PDCs isolated either through enzymatic digestion or egression of PDCs from resected periosteum 3 5 5, 13. The inner cambium layer is composed of multiple cell types, with a significant subpopulation (>99%) of cambial cells identified as mesenchymal stem cells or periosteum‐derived stem cells (PDCs) 3, 5. Hence, the cambium layer can be seen as a putative MSC and osteochondral progenitor cell niche 1, 2, 7, 14 15 16 17. In the present study, Hoechst nuclear stain was used to identify cells in the cambium layer. In addition, our current live imaging method is limited to population‐based study of PDCs; however, future studies might enable single cell study of nuclear shape changes associated with loss in periosteal tissue's endogenous stress and changes in collagen crimping. To date, such single cell‐based studies are limited to live cells in culture or fixed tissues; a shortcoming of studying fixed cells is the anisotropic shrinking of the extracellular matrix constituents and cells induced by chemical fixation 38.

A great impetus exists for elucidation of periosteum mechanobiology in the context of regenerative medicine 6, 11. A number of in vivo preclinical studies have shown PDCs to be comparable or superior to bone marrow stem cells for bone healing and regeneration 3, 58 59 60. In addition, recent studies of PDCs isolated from the periosteum of hip replacement patients have shown PDCs to have in vitro bone and cartilage generation capacity comparable to that of commercially available human bone marrow stromal cells 3. PDCs isolated either by enzymatic digestion or migration exhibit subtle differences in the expression of surface markers typical for MSCs but no difference in proliferation or multipotency. Human PDCs have been better characterized than ovine PDCs, because the cell surface markers are commercially available for flow cytometry analysis, in addition to primers being widely available for the assessment of changes in gene transcription 2, 3, 61 62 63 64 65. Hence, a limiting factor of our study was that, to date, the PDC surface markers available for human cells are not yet available for ovine cells. However, previous studies have demonstrated that ovine periosteal‐derived cell morphological patterns of phenotype remain the same as those for human PDCs 2, 3. Although we ultimately aim to translate the current approach to the human condition, access to sufficient quantities of fresh human periosteum with and without prestress is currently not ethically or practically feasible, although cutting edge imaging technologies enabling seamless imaging from the organ to the nano‐length scale promise an exciting future in this regard 66, 67. Quantitative characterization of ovine periosteum as a stem cell niche provides a critical step in the clinical translation of periosteum's regenerative power in the context of the ovine model and its recent application to limited human patients 25. Important next steps include quantification of cell numbers and proliferation throughout the periosteal thickness, in both the cambium and the fibrous layers (e.g., through quantification of DNA). Also, as surface markers for cell cytometry and primers for quantitative polymerase chain reaction become available for ovine cells and tissues, it will be important to link our current understanding of human PDCs obtained during the course of hip replacement surgery 2, 3 to our understanding of periosteum modulated tissue generation in a well‐established and controlled ovine femur segmental defect model 4, 5, 9, 10, 13, 18.

## Conclusion

Using a novel live cell and tissue imaging protocol, we have demonstrated quantitatively that the loss of the periosteum's prestress coincides with a significant increase in nuclear rounding and changes in the architecture of ECM structural proteins of the stem cell niche. Taken together, these and previous studies provide a new appreciation for the role of mechanics in the modulation of periosteal stem cell niche quiescence.

## Author Contributions

N.Y.C.Y.: conception and design, collection and/or assembly of data, data analysis and interpretation, manuscript writing, final approval of manuscript; C.A.O.: data analysis and interpretation; I.S.: conception and design, collection and/or assembly of data, final approval of manuscript; R.M.W.: conception and design, collection and/or assembly of data, final approval of manuscript; M.L.K.T.: conception and design, data analysis and interpretation, manuscript writing, final approval of manuscript.

## Disclosure of Potential Conflicts of Interest

M.L.K.T. has uncompensated employment and intellectual property rights. The other authors indicated no potential conflicts of interest.

## Supporting information

Supporting Information
