# Supplementary material for: Live Tissue Imaging to Elucidate Mechanical Modulation of Stem Cell Niche Quiescence
Source: Stem Cells Transl Med. 2016 Jul 28;6(1):285–92. doi: 10.5966/sctm.2015-0306 (PMC5442759; doi:10.5966/sctm.2015-0306)
Supplement: Supplementary file 1 — Supporting Information [file SCT3-6-285-s001.pdf]

Supplemental Figure – Knothe Tate et al.

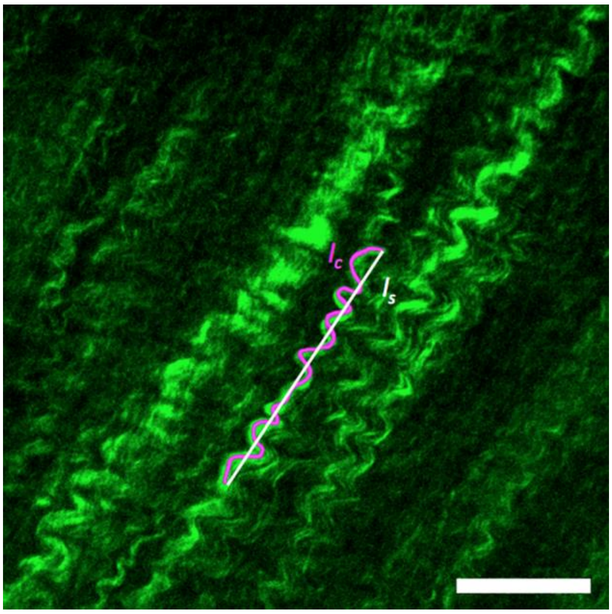

Figure S1. Representative image demonstrating definition of measured parameters for collagen crimp measurement. The curved length of collagen fiber bundles (l_c) and straight line (l_s) connecting the ends of measured fiber bundle were measured with semi-automated NeuroJ plugin (ImageJ2), giving the degree of crimp in collagen filaments ***C= (lc-ls)/ls x 100%.***
